# Cost analysis of open versus robot-assisted ventral hernia repair – a retrospective cohort study

**DOI:** 10.1007/s10029-024-03089-7

**Published:** 2024-06-26

**Authors:** Nadia A. Henriksen, Mads Marckmann, Mette Willaume Christoffersen, Kristian K. Jensen

**Affiliations:** 1 Dept. of Gastrointestinal and Hepatic Diseases, Herlev Hospital, University of Copenhagen, Borgmester Ib Juuls Vej 1, Herlev, DK-2730 Denmark; 2 Digestive Disease Center, Bispebjerg Hospital, Copenhagen, Denmark; 3 Dept. of Surgery, Zealand University Hospital, Koege, Denmark; 4 Dept. of Surgery and Transplantation, Rigshospitalet, Copenhagen, Denmark

**Keywords:** Incisional hernia, Umbilical hernia, Readmission, Length of stay

## Abstract

**Background:**

Robot-assisted ventral hernia repair is associated with decreased length of stay and lower complication rates compared with open repair, but acquisition and maintenance of the robotic system is costly. The aim of this was study was to compare the procedure-specific cost of robot-assisted and open ventral and incisional hernia repair including cost of procedure-related readmissions and reoperations within 90 days postoperatively.

**Methods:**

Single-center retrospective cohort study of 100 patients undergoing robot-assisted ventral hernia. Patients were propensity-score matched 1:1 with 100 patients undergoing open repairs on age, type of hernia (primary/incisional), and horizontal defect size. The primary outcome of the study was the total cost per procedure in Euros including the cost of a robotic approach, extra ports, mesh, tackers, length of stay, length of readmission, and operative reintervention. The cost of the robot itself was not included in the cost calculation.

**Results:**

The mean length of stay was 0.3 days for patients undergoing robot-assisted ventral hernia repair, which was significantly shorter compared with 2.1 days for patients undergoing open repair, *P* < 0.005. The readmission rate was 4% for patients undergoing robot-assisted ventral hernia repairs and was significantly lower compared with open repairs (17%), *P* = 0.006. The mean total cost of all robot-assisted ventral and incisional hernia repairs was 1,094 euro compared with 1,483 euro for open repairs, *P* = 0.123. The total cost of a robot-assisted incisional hernia repair was significantly lower (1,134 euros) compared with open ventral hernia repair (2,169 euros), *P* = 0.005.

**Conclusions:**

In a Danish cohort of patients with incisional hernia, robot-assisted incisional hernia repair was more cost-effective than an open repair due to shortened length of stay, and lower rates of readmission and reintervention within 90 days.

## Introduction

Ventral hernia repair is one of the most frequently performed elective surgeries worldwide. The surgical approaches vary substantially depending on the patient characteristics, type, and location the of ventral hernia, as well as the surgeons’ preferences and equipment availability. Hence, a small primary ventral hernia may be repaired in a day case setting with open sutured or mesh repair, whereas an incisional hernia repair is more complex possibly requiring preoperative patient optimization, a more technically challenging repair, and postoperative hospital stay.

The choice between an open or minimally invasive approach has been debated for years, and it seems clear that the risk of wound infection is significantly decreased when choosing a minimally invasive approach, whereas there are no significant difference in recurrence rates [1–4]. The use of mesh to decrease recurrence is undebatable, and it is optimally placed in the preperitoneal or retromuscular plane for umbilical and incisional hernias, respectively [5, 6].

In the past decades, the use of a robotic system for ventral hernia repair has gained increasing popularity due to an easy access to the preperitoneal or retromuscular plane with a minimally invasive technique. The first data suggested that robot-assisted ventral hernia repair decreased the length of postoperative hospital stay significantly [7]. Two recent nationwide cohort studies confirmed these findings and further concluded that postoperative morbidity was decreased for robot-assisted repairs compared to open or laparoscopic ventral hernia repairs [8, 9]. Specifically, readmission rates were significantly decreased after robot-assisted repair compared with laparoscopic ventral hernia repair, whereas both readmission and the need for surgical reintervention were decreased when compared to open ventral hernia repair.

However, critics of robot-assisted surgery argue that it is time-consuming, too expensive, and that further cost-effectiveness analyses are needed to weigh the clinical benefits [10–12]. Acquisition and maintenance of the robotic system is costly, but data on procedure-specific cost of robot-assisted ventral hernia repair is inadequately investigated. Hypothetically, the decreased length of stay, readmission, and reintervention rates may offset the cost of the robotic system.

This single-center retrospective analysis was undertaken to compare the procedure-specific cost of robot-assisted with open primary ventral and incisional hernia repair, including cost of procedure-related readmissions and reinterventions within 90 days postoperatively.

## Materials and methods

This was a retrospective single-center cohort study of patients undergoing primary ventral and incisional hernia repair at a University Hospital in Copenhagen, Denmark. Patients eligible for inclusion in the study were identified in a local prospective database of all ventral hernia procedures. The study period was from January 1st, 2017, to January 1st, 2023.

To compare the costs associated with open and robot-assisted ventral hernia repair, patients undergoing robot-assisted repair were matched in a 1:1 ratio with patients undergoing open repair, thus reducing the risk of bias in the results.

Variables included in the study were type of approach (robot-assisted/open), age, sex, American Society of Anesthesiologist (ASA) score, tobacco smoking, diabetes, cardiac disease, pulmonary disease, Body Mass Index (BMI), horizontal and vertical fascial defect size, type of procedure performed, including the use of component separation, and type of mesh used. Postoperative variables included length of hospital stay, 90-day readmission rate (including number of days readmitted), and number of surgical reinterventions within 90 days.

Patients underwent hernia repair in the same time period and were discharged using same discharge criteria [13]. Patients were scheduled for type of repair (open/robotic) based on patient characteristics and the attending surgeons’ preferences. Readmission criteria were the same for the two groups and decided by patients’ general practitioner or at the emergency ward.

### Cost analysis

The primary outcome of the study was the total mean cost per procedure given in Euro. The cost analysis was performed on the differences in cost per procedure, as both open and robot-assisted procedures involved an equal number of staff (nurses and surgeons) and had comparable expenses regarding anesthesia and procedural instruments. Thus, the following cost variables were included: Cost of a robot-assisted approach, extra ports (in case of more than three ports used), mesh, tackers, hospital length of stay, length of readmission, and operative reintervention. The cost of a robot-assisted approach was calculated as the price per procedure including instrument arm drape, one monopolar and one bipolar cable, three instrument cannulas including obturators, cannula seal and tip covers, and one of each of the following instruments: monopolar curved scissor, fenestrated bipolar forceps and mega suture cut needle driver. The cost associated with purchasing the robotic system was not included. The cost per day in hospital (postoperatively and in case of readmission within 90 days) was retrieved using the national Diagnosis Related Grouping (DRG) rate, which is a measure of the hospital costs associated with hospital procedures. The same system was used to estimate the cost of operative reintervention, using the DRG rate “Other operations and treatments on digestive organs without complicated secondary diagnoses” to not overestimate the costs. The same price was used for every surgical reintervention.

### Statistics

To establish the cohort, the first 100 patients undergoing robot-assisted hernia repair in the study period were matched in a 1:1 ratio with patients undergoing open repair using propensity score matching. Patients were matched on the confounding variables age, type of hernia (primary/incisional) and horizontal defect size using nearest neighbor matching with a caliper of 0.05. Numerical variables were reported as mean (standard deviation, SD) or median (range) and compared across the two groups of patients using Student’s t-test or Mann–Whitney U test where appropriate. Categorical variables were reported as n (%) and compared across groups using the Chi-squared test. To examine the association between mean total costs per procedure and the confounding variables a linear regression analysis was performed. All statistical analyses were performed on the entire cohort and in subgroup analyses of patients undergoing primary or incisional hernia repair, respectively.

P-values < 0.05 were considered statistically significant. The data analysis was performed using R software version 4.0.2 (R Foundation for Statistical Computing, Vienna, Austria).

This study was approved by the Danish Data Protection Agency (ref. P-2021-58). All patients gave written consent to chart review but were otherwise not included in the research process. A protocol was made before study start, but not published online. The study was registered at ClinicalTrials.gov identifier: NCT06232148. The study is reported according to the STROCSS 2021 guidelines for cohort studies [14].

## Results

**Fig. 1:**
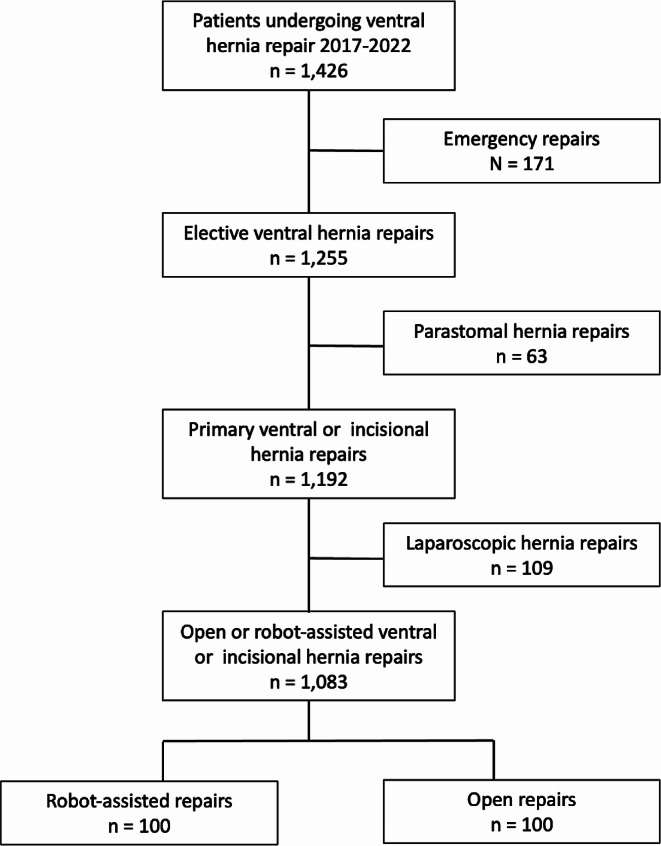
Flowchart of patient inclusion

A total of 1,083 patients underwent robot-assisted or open ventral hernia repair from 2017 to 2022 (Fig. 1). A total of 100 patients undergoing robot-assisted ventral hernia repair were matched with 100 patients undergoing open repair. There were 37 patients with primary ventral hernias and 63 patients with incisional hernias in each group. Significantly more patients in the robot-assisted repair group were active smokers and had higher BMI (Table 1).

**Table 1:** Demographics of patients undergoing robot-assisted and open ventral hernia repair

		Robot-assisted approach (*n*=100)	Open repair (*n*=100)	*P*
Age (years)	*<45*	14 (14.0)	16 (16.0)	0.979
	*45-60*	39 (39.0)	39 (39.0)	
	*>60-75*	35 (35.0)	34 (34.0)	
	*>75*	12 (12.0)	11 (11.0)	
Female gender		40 (40.0)	45 (45.0)	0.567
ASA score	*I* *II* *III*	36 (36.0) 46 (46.0) 18 (18.0)	46 (46.0) 40 (40.0) 14 (14.0)	0.343
Smoking	*Yes*	35 (35.0)	9 (9.0)	< 0.001
Diabetes	*Yes*	14 (14.0)	7 (7.0)	0.166
Cardiac disease	*Yes*	17 (17.0)	15 (15.0)	0.847
Pulmonary disease	*Yes*	16 (16.0)	18 (18.0)	0.851
BMI (kg/m^2^)	*mean [range]*	31 [18.4, 44.6]	28.7 [17.1, 42.0]	0.003
Type of hernia	*Primary*	37 (37.0)	37 (37.0)	
	*Incisional*	63 (63.0)	63 (63.0)	>0.99
Vertical defect size (cm)	*mean (sd)*	6.8 (5.5)	6.5 (5.9)	0.630
Horizontal defect size (cm)	*mean (sd)*	4.9 (3.2)	4.8 (3.3)	0.872
Approach	*TARUP*	64 (64.0)	0 (0.0)	< 0.001
	*RoboTAR*	20 (20.0)	0 (0.0)	
	*TAPP*	7 (7.0)	0 (0.0)	
	*Retromuscular*	1 (1.0)	0 (0.0)	
	*eTEP*	8 (8.0)	0 (0.0)	
	*Open*	0 (0.0)	100 (100.0)	
Component separation	*No*	72 (72.0)	85 (85.0)	0.002
	*Unilateral TAR*	10 (10.0)	0 (0.0)	
	*Bilateral TAR*	18 (18.0)	12 (12.0)	
	*Bilateral ECS*	0 (0.0)	3 (3.0)	
Mesh position	*Onlay*	0 (0.0)	10 (10.0)	< 0.001
	*Preperitoneal*	7 (7.0)	19 (19.0)	
	*Retromuscular*	93 (93.0)	54 (54.0)	
	*Intraperitoneal*	0 (0.0)	17 (17.0)	
Type of mesh	*Parietex Progrip*	83 (83.0)	46 (46.0)	< 0.001
	*Vypro, Ethicon*	0 (0.0)	1 (1.0)	
	*Versatex*	0 (0.0)	8 (8.0)	
	*Ventralex ST, BARD*	0 (0.0)	35 (35.0)	
	*Parietene Composite*	0 (0.0)	2 (2.0)	
	*Bard Softmesh*	17 (17.0)	6 (6.0)	
	*Adhesix, BARD*	0 (0.0)	1 (1.0)	
	*Galmesh Light*	0 (0.0)	1 (1.0)	
Length of stay (days)	*mean [range]*	0 [0, 3]	2.1 [0, 19]	< 0.001
Readmission, n (%)	*Yes*	4 (4.0)	17 (17.0)	0.006
Reoperation, n (%)	*Yes*	1 (1.0)	8 (8.0)	0.041

*SD*: Standard deviation. *BMI*: Body Mass Index. *TAPP*: Transabdominal preperitoneal prosthesis repair. *RoboTAR*: Robotic Transversus Abdominis Release. *TARUP*: Robotic transabdominal retromuscular umbilical prosthetic hernia repair. *eTEP*: Extended totally extraperitoneal repair. *TAR*: Transversus Abdominis Release. *ECS*: Endoscopic Component Separation

In the group of patients undergoing robot-assisted repair, the vast majority had the mesh placed in the retromuscular plane (93/100) using the Transabdominal Retromuscular Umbilical Prosthetic (TARUP) approach (64/100). In the group of patients undergoing open repair, the mesh placement was in the retromuscular plane in approximately half of the cases (54/100), followed by the preperitoneal (19/100), intraperitoneal (17/100), and onlay plane (10/100) (Table 1). A self-gripping mesh was the most used in both groups, though it was used significantly more in the robot-assisted repairs (83/100) compared with the open repairs (46/100, *P* < 0.005).

The mean length of stay was 0 days for patients undergoing robot-assisted ventral hernia repair, which was significantly shorter compared with 2.1 days for patients undergoing open repair, *P* < 0.005 (Table 2). The readmission rate was 4% for robot-assisted ventral hernia repairs, which was significantly lower compared with open repairs (17%), *P* = 0.006 (Table 2). Only 1% of the patients in the robot-assisted repair group underwent a surgical reintervention within 90 days compared with 8% in the open group, *P* = 0.041 (Table 1).

**Table 2:** Mean total cost per patient for robot-assisted and open ventral and incisional hernia repair

		Robot-assisted approach (*n*=100)	Open repair (*n*=100)	*P*
*Costs (Euros)*				
*Robotic approach, Euro*	*mean (sd)*	881.4 (0)	0 (0)	< 0.001
*Extra ports, Euro*	*mean (sd)*	12.9 (27.7)	0 (0)	< 0.001
*Mesh, Euro*	*mean (sd)*	129.2 (0.3)	155.6 (44.2)	< 0.001
*Tackers, Euro*	*mean [sd]*	0 (0)	7 (40.1)	0.082
*Length of stay, Euro*	*mean (sd)*	8.7 (87.4)	617.3 (964.7)	< 0.001
*Readmission, Euro*	*mean (sd)*	23.3 (128.8)	276.6 (1,070)	0.019
*Reoperation, Euro*	*mean (sd)*	38.7 (387.5)	426.2 (1,547.5)	0.015
***Total, Euro***	*mean (sd)*	1,094.3 (512.7)	1,482.9 (2,468.9)	0.123

Of the patients undergoing surgical reintervention in the open group, 5% had one reoperation, and 3% had two reoperations. The surgical reinterventions were reoperation for deep infection (2%) or superficial bleeding (2%), exploratory laparotomy (1%), suture of fascial dehiscence with traction mesh (1%), excision of pathological tissue (1%) and other reoperation after gastrointestinal surgery (1%). The one surgical reintervention in the robot-assisted group was an exploratory laparotomy.

The mean total cost of robot-assisted ventral repairs was 1,094 euro compared with 1,483 euro for open repairs, *P* = 0.123 (Table 2). In multivariable logistic regression analysis adjusted for age, gender, ASA score, BMI, defect size and type of hernia (primary ventral/incisional), incisional hernia repair, BMI > 35 kg/m^2^ and horizontal defect size > 4 cm were factors significantly associated with increased total costs, whereas robot-assisted repair was significantly associated with decreased total costs (*P* = 0.010), (Table 3).

**Table 3:** Multivariable analysis of factors associated with total costs for patients operated for primary ventral and incisional hernia

		coefficient	95% CI	*P*
Age *(years)*		-12.81	[-31.33;5.70]	0.178
Sex	*Female*	Ref		
	*Male*	438.13	[-22.28;898.54]	0.064
ASA score	*I*	Ref		
	*II*	456.20	[-206.45;1118.85]	0.179
	*III*	325.09	[-493.18; 1143.37]	0.437
BMI	*< 30*	Ref		
	*30-35*	135.58	[-521.40;*792.55]*	0.686
	*> 35*	861.14	[51.08;1671.20]	0.039
Horizontal defect, cm	*<4*	Ref		
	*4-8*	1241.85	[640.83;1842.87]	<0.001
	*>8*	825.56	[112.03;1539.09]	0.024
Robot-assisted approach	*yes*	-602.12	[-1055.56;-148.69]	0.010
Type of hernia	*Primary*	Ref		
	*Incisional*	696.90	[139.06;1254.74]	0.015

In a subgroup analysis including primary ventral hernias only, there were no significant differences between robot-assisted and open approach on length of stay (0 vs. 0.4 days, *P* = 0.149) and readmission (5.4% vs. 2.7%, *P* = 0.556). None of the patients who underwent primary ventral hernia repair required surgical reintervention. The mean total cost of a robot-assisted primary ventral hernia repair was significantly higher (1,026 euros) compared with open primary ventral hernia repair (315 euros), *P* < 0.001, (Table 4).

**Table 4:** Mean total cost per patient for robot-assisted and open primary ventral hernia repair

			Robot-assisted approach (*n*=37)		Open repair (*n*=37)	*P*
*Costs (Euros)*						
*Robotic approach, Euro*	*mean (sd)*	881.4 (0)		0 (0)		< 0.001
*Mesh, Euro*	*mean (sd)*	129.1 (0)		176 (50.8)		< 0.001
*Tackers, Euro*	*mean [sd]*	0 (0)		12.6 (53.6)		0.154
*Length of stay, Euro*	*mean (sd)*	0 (0)		118.1 (497.1)		0.149
*Readmission, Euro*	*mean (sd)*	15.7 (66.8)		7.9 (47.9)		0.558
***Total, Euro***	*mean (sd)*	1,026.2 (66.8)		314.6 (488.4)		< 0.001

In a subgroup analysis including incisional hernias only, the length of stay was significantly longer for open repairs (3.1 days) compared to robot-assisted repairs (0 days), *P* < 0.001. The rates of readmission and surgical reintervention were significantly decreased for robot-assisted repairs (3.2% and 1.6%) compared with open repairs (25.4% and 12.7%), *P* = 0.001 and *P* = 0.038, respectively. The total cost of a robot-assisted incisional hernia repair was significantly lower (1,134 euros) compared with open incisional hernia repair (2,169 euros), *P* = 0.005, (Table 5). In multivariable logistic regression analysis adjusted for age, gender, ASA score, BMI and defect size, robot-assisted repair was significantly associated with decreased total costs (*P* < 0.001), (Table 6).

**Table 5:** Mean total cost per patient for robot-assisted and open incisional hernia repair

		Robot-assisted approach (*n*=63)	Open repair (*n*=63)	*P*
*Costs (Euros)*				
*Robotic approach, Euro*	*mean (sd)*	881.4 (0)	0	< 0.001
*Extra ports, Euro*	*mean (sd)*	20.5 (32.6)	0	< 0.001
*Mesh, Euro*	*mean (sd)*	129.3 (0.3)	143.7 (35.1)	0.001
*Tackers, Euro*	*mean (sd)*	0 (0)	3.7 (29.5)	0.317
*Length of stay, Euro*	*mean (sd)*	13.9 (110.1)	910.6 (1,051.9)	< 0.001
*Readmission, Euro*	*mean (sd)*	27.7 (154.4)	434.5 (1,326.1)	0.016
*Reoperation, Euro*	*mean (sd)*	61.5 (488.2)	676.6 (1,911)	0.013
***total, Euro***	*mean (sd)*	1,134.3 (642.4)	2,169 (2,881.2)	0.005

**Table 6:** Multivariable analysis of factors associated with total costs for patients operated for incisional hernia

		coefficient	95% CI	*P*
Age *(years)*		-24.20	[-54.11;5.71]	0.035
Sex	*Female*	Ref		
	*Male*	293.50	[-383.20;970.19]	0.397
ASA score	*I*	Ref		
	*II*	926.77	[-44.24;1897.19]	0.064
	*III*	877.99	[-265.48;2021.46]	0.135
BMI	*< 30*	Ref		
	*30-35*	-125.73	[-1069.85;818.38]	0.795
	*> 35*	1360.96	[127.95;2593,96]	0.032
Horizontal defect, cm	*<4*	Ref		
	*4-8*	1528.02	[738.65;2317.40]	<0.001
	*>8*	1203.86	[327.12;2080.60]	0.008
Robot-assisted approach	*yes*	-1175.01	[-1839.38;-510.64]	<0.001

## Discussion

The mean cost of a robot-assisted incisional hernia repair including inpatient hospital cost, readmissions, and surgical reinterventions within 90 days was significantly lower compared with open incisional hernia repair. The significant cost reduction in the robot-assisted repair was due to a more than halved length of stay, 4-fold decreased readmissions, and reoperations for patients undergoing incisional hernia repair. When including both primary ventral and incisional hernia repairs, the cost of open and robot-assisted ventral hernia repair was comparable.

A significant cost reduction of robot-assisted abdominal wall reconstruction compared to an open approach including equipment and inpatient hospital stay was also reported in a recent study from Canada [15]. This cost reduction was driven by a shorter inpatient hospital stay for patients receiving a robot-assisted repair. Another recently published study from the US including 1,300 patients reported that the hospital costs were higher for robot-assisted repairs, however, the complications rates were lower, resulting in comparable costs of open and robot-assisted repairs when the post-discharge expenses were included [16]. In a meta-analysis on costs and outcomes after robot-assisted and open ventral hernia repair, it was reported that the overall costs were higher for the robot-assisted approach, but emphasized that for large complex hernias, the robotic platform may be an effective minimally invasive approach with a clinical benefit that could potentially offset the costs [12]. Conclusively, existing data suggests that the use of robot-assisted repair for incisional hernias and more complex abdominal wall reconstruction is cost beneficial compared to open repairs due to significantly shortened length of stay and less postoperative complications.

Interestingly, no cost benefit of robotic platform was found when analyzing the repair of primary ventral hernias separately. Open repair of a primary ventral hernia is often less complicated than an open incisional hernia repair, where patients often have more postoperative pain, which may increase length of stay. This is an important finding, as one may argue that the robot should only be used in patients where there is a clinical and economic benefit. However, this depends on the hospital system and the access to robot at a given site. Furthermore, there were more patients that were active smokers and with high BMI in the robotic group, both of which are risk factors for wound complications even in patients with small umbilical hernias [17]. Decreasing wound complications may further reduce long-term recurrence, which could potentially extend the cost-beneficial effect of the robotic platform in both primary ventral and incisional hernia repair.

Health-cost and expenses of surgical procedures vary greatly in public and private sectors and across countries, which may impede comparison. In the present study, the entire cost was calculated including surgical equipment including expenses of length of stay, readmission, and surgical reintervention. It was decided to use one fixed minimum cost for a surgical reintervention, even though some procedures could potentially have been more expensive than others, meaning that equipment used for the surgical reintervention was not calculated. Cost of personnel was not calculated, but there was no reason to believe that this differed between groups. A cost calculation naturally depends on what is included. One may argue that acquisition of the robotic platform is considerably expensive and should be included in a cost calculation. On the contrary, the investment in a robotic platform is a part of innovation and adaptation of new technologies and the process may be compared to the implementation era of laparoscopic surgery.

The study is limited by the fact that it is a smaller retrospective case series. The cost analysis did not include acquisition of the robotic platform or operating times, which would have increased the costs in the robotic group. On the other hand, minimum prices were used for days in hospital and surgical reinterventions to be pragmatic. If imaging diagnostics or epidural was required, it was not included in the cost calculation. Furthermore, specific surgical equipment such as vacuum assisted closure (VAC), staplers or energy devices were not included in the price of the surgical reintervention, which potentially could have increased the costs in the open procedure group. Matching was performed on age, defect size and type of hernia. One could argue that ASA score or comorbidity should have been included as potential confounders. However, there was tendency towards choosing a robotic approach for patients with highest BMI and more comorbidity, why using these factors as matching variables could potentially have resulted in a wrong picture with even higher cost in the open group. Nevertheless, ASA score and BMI was included in the multivariable analyses. Furthermore, it could be suggested that the robotic approach should be compared to a laparoscopic approach, instead of open repair, as two minimally invasive techniques are more comparable. However, we have found in another study that the length of stay and readmission rate is longer for conventional laparoscopic ventral hernia repair compared with a robotic approach [8]. However, further studies are needed to clarify the cost-effectiveness of laparoscopic and robotic ventral hernia repair.

In conclusion, the current study evaluated procedure specific costs of robot-assisted and open ventral hernias repairs without including the acquisition cost of the robotic system in a cohort of patients in Denmark. Open primary ventral hernia repair was more cost effective than robot-assisted repair, but robot-assisted incisional hernia repair was more cost effective than an open repair due to shortened length of stay, and lower rates of readmission and reintervention within 90 days.
